# Real-time monitoring of laser powder bed fusion process using high-speed X-ray imaging and diffraction

**DOI:** 10.1038/s41598-017-03761-2

**Published:** 2017-06-15

**Authors:** Cang Zhao, Kamel Fezzaa, Ross W. Cunningham, Haidan Wen, Francesco De Carlo, Lianyi Chen, Anthony D. Rollett, Tao Sun

**Affiliations:** 1 X-ray Science Division, Advanced Photon Source, Argonne National Laboratory, Argonne, IL 60439 USA; 2 Department of Materials Science and Engineering, Carnegie Mellon University, Pittsburgh, PA 15213 USA; 3 Department of Mechanical and Aerospace Engineering, Missouri University of Science and Technology, Rolla, MO 65409 USA

## Abstract

We employ the high-speed synchrotron hard X-ray imaging and diffraction techniques to monitor the laser powder bed fusion (LPBF) process of Ti-6Al-4V *in situ* and in real time. We demonstrate that many scientifically and technologically significant phenomena in LPBF, including melt pool dynamics, powder ejection, rapid solidification, and phase transformation, can be probed with unprecedented spatial and temporal resolutions. In particular, the keyhole pore formation is experimentally revealed with high spatial and temporal resolutions. The solidification rate is quantitatively measured, and the slowly decrease in solidification rate during the relatively steady state could be a manifestation of the recalescence phenomenon. The high-speed diffraction enables a reasonable estimation of the cooling rate and phase transformation rate, and the diffusionless transformation from *β* to *α* ^*’*^ phase is evident. The data present here will facilitate the understanding of dynamics and kinetics in metal LPBF process, and the experiment platform established will undoubtedly become a new paradigm for future research and development of metal additive manufacturing.

## Introduction

Additive manufacturing (AM, a.k.a. 3D printing) of metallic materials has been witnessing tremendous growth over the past three decades^1–5^, particularly in the fields of medical, aerospace, automobile, and defense industries^6, 7^. Currently, most of the metal AM systems are of the laser powder bed fusion (LPBF) type owing to its superior capability to make geometrically complex parts^8, 9^. In a typical LPBF process, a laser beam scans across a thin layer of metallic powders, and locally melts the powders through to the layer below^10, 11^. Although the process is conceptually simple, there are many highly dynamic and transient physical phenomena involved because of the extremely high heating and cooling rates^12–15^, e.g. melting and partial vaporization of metallic powders, flow of the molten metal, powder ejection and re-distribution, rapid solidification, non-equilibrium phase transition, etc. Oftentimes, the complex interactions result in a product with rough surface, significant porosity in terms of both size and number density, residual stress, and unfavorable phase and grain structures, with the consequent impact on properties^16–19^. In order to understand the mechanisms responsible for the formation of these defects, it is imperative to develop and apply *in situ* characterization techniques to study the dynamic microstructural evolution^18, 20, 21^. Unfortunately, it is extremely challenging to experimentally characterize the dynamics of the LPBF process due to the highly localized and very fast interaction of the laser beam with metal powders. The most advanced characterization method reported so far that uses a high-speed visible light imaging, can only monitor what happens above the surface of the powder bed^15^.

Here, we report *in situ* probing the dynamics of the LPBF process inside as well as above the surface of the powder bed using high-speed hard X-ray imaging and diffraction techniques at the Advanced Photon Source (APS). It is demonstrated that quantitative structural information on melt pool size/shape, powder ejection, solidification, and phase transformation can be obtained from the high-resolution time-resolved X-ray images and diffraction patterns. The experimental and data analysis approaches developed here will provide a compass pointing to the fundamental understanding of the physics in the AM process, and will further accelerate the coming of the AM age.

## Results and Discussion

### High-speed synchrotron X-ray imaging and diffraction

**Figure 1:**
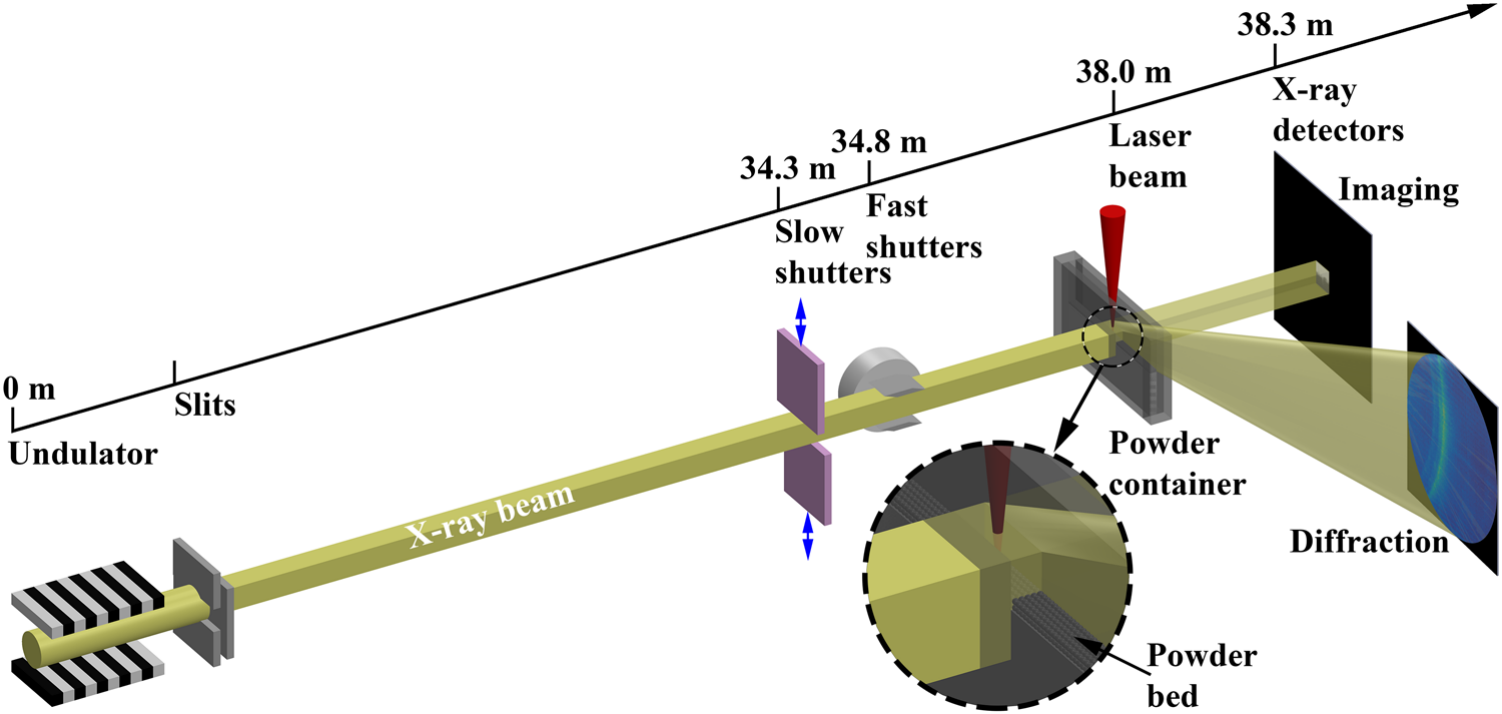
Schematic of the high-speed X-ray imaging and diffraction experiments on laser powder bed fusion process at the 32-ID-B beamline of the Advanced Photon Source. A short-period undulator generates a pseudo pink beam with first harmonic energy of 24.4 keV (*λ* = 0.508 Å). The laser impinges on the miniature powder bed sample from the top, and the X-rays penetrate the sample from the side. The imaging and diffraction detectors are placed downstream, about 300 mm away from the sample. The inset surrounded by the dashed circle enlarges the view of the laser-sample and X-ray-sample interaction. The distance of each component from the source is labeled on top.

The high-speed X-ray imaging and diffraction experiments were carried out at the 32-ID-B beamline of the APS^22–25^. The schematic of the synchrotron X-ray experiments is depicted in Fig. 1. A short-period (1.8 cm) undulator with the gap set to 12 mm generates polychromatic X-rays with the first harmonic energy at 24.4 keV and the corresponding wavelength at 0.508 Å. A pair of slits was used to define the size of the X-ray beam. For imaging experiments, the dimensions of the slit window were typically set to 1 mm × 1 mm, which yields an integrated flux of ~7 × 10^15^ ph/s. Since the photon flux with higher harmonic energies is relatively low (less than 4% of overall flux as the energy spectrum shown in Supplementary Fig. 1), the incident beam behaves similarly as a pink beam with an energy bandwidth of ~4%. In diffraction experiments, the slits were further closed to reduce the energy bandwidth. At the beamline, a set of slow shutters and a set of fast shutters were employed to define a very small time window for the X-rays to pass through, in order to protect the detection system. The samples were located at about 38 m away from the source. The high-speed imaging camera was placed 310 mm away from the sample in the downstream for recording full-field X-ray images, while the high-speed diffraction detector had an angle offset of 14.6° from the incident beam and the sample-to-detector distance was 235 mm. The samples studied in the experiments were miniature Ti-6Al-4V powder bed systems, which consisted of two pieces of glassy carbon plates (1 mm thickness, and 6 mm height) as the container walls, one piece of Ti-6Al-4V plate (450 µm thickness, 3 mm height) sandwiched between the carbon plates as the base, and one layer of Ti-6Al-4V powders (~100 µm thickness) evenly spread out on top of the metal base. The powder bed samples were at room temperature before laser heating, and no preheating was applied. The powder used in this study was EOS *Titanium Ti64* powder (Ti-6Al-4V alloy, particle diameter < 60 µm), designed for use in the commercially available EOS LPBF systems. The metal samples were located inside a stainless steel chamber with argon protection atmosphere. The laser beam carrying various powers entered into the chamber straight down with a spot size of ~220 µm (1/e^2^, Gaussian beam) on the powder bed. The laser was operated in a spot-heating mode with no scanning involved. The X-ray beam impinged on the samples horizontally providing a side view of the powder bed. A series of delay generators were used to synchronize the shutters, laser, and X-ray detectors. More details of the experimental setup are described in the “Methods” section and Supplementary Fig. 2.

### Real-time monitoring of the laser powder bed fusion process

**Figure 2:**
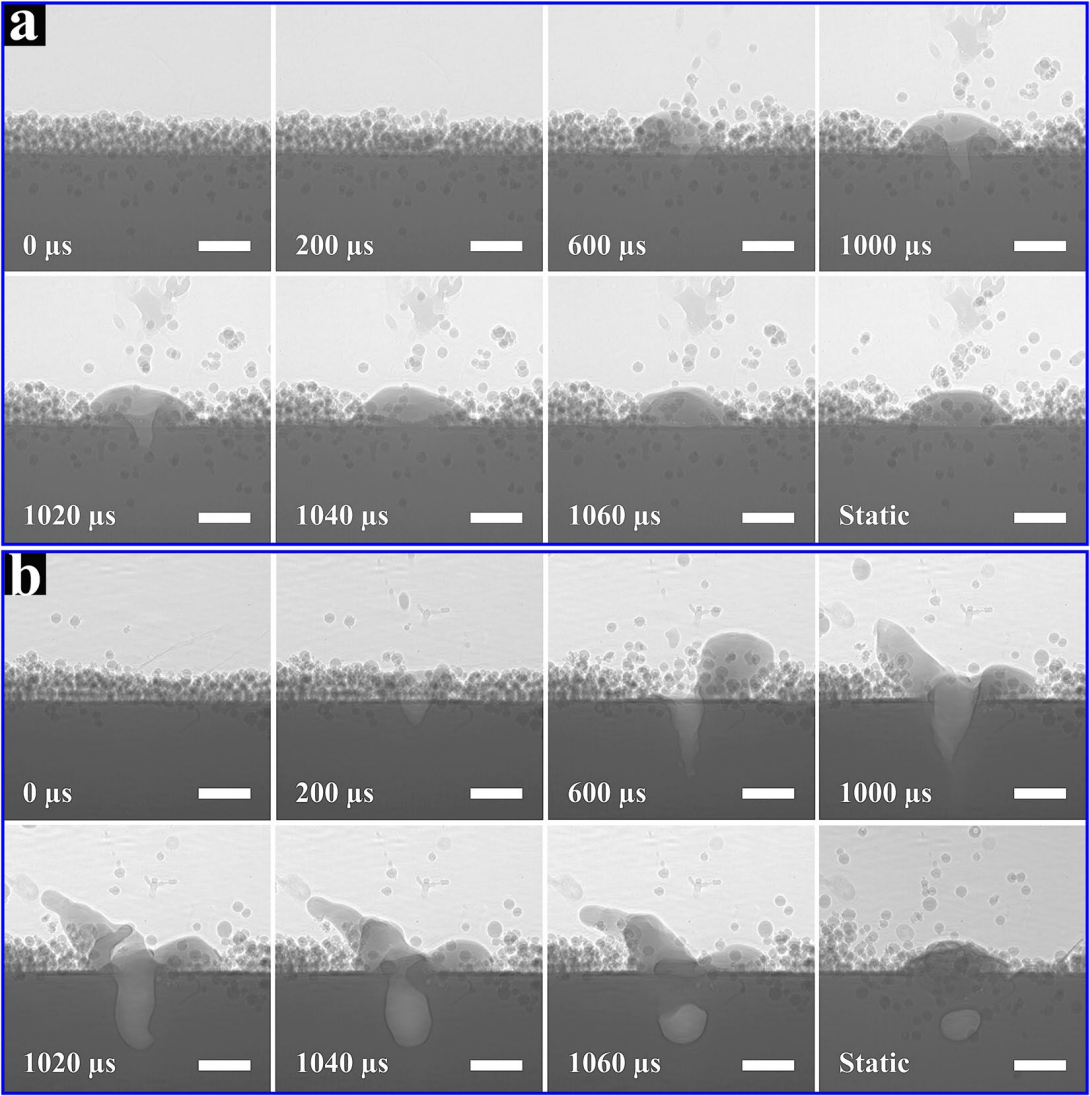
Dynamic X-ray images of laser powder bed fusion processes of Ti-6Al-4V. The laser powers are 340 W for image group (**a**) and 520 W for group (**b**), respectively. The laser beam size is ~220 µm (1/e^2^). The powder particle size is in the range of 5–45 µm, and the powder layer thickness is ~100 µm. The numbers indicate the time nodes. The laser is turned ON at *t* = 0, and continues to heat the sample till *t* = 1000 µs. The raw data were taken with a frame rate of 50 kHz. The exposure time for each individual image is 350 ns. All the scale bars are 200 µm.

High-speed X-ray images of LPBF processes with two different laser power conditions are shown in Supplementary Videos 1 and 2. Both image series were taken with a frame rate of 50 kHz and an exposure time of 350 ns for each individual image. Figure 2 displays eight X-ray images of each series. In Fig. 2a and Supplementary Video 1, the laser power and duration time were set to 340 W and 1 ms, respectively. It can be observed that the process starts with the melting of the Ti-6Al-4V powders around the laser spot and then the Ti-6Al-4V base. Subsequently, a small cavity or depression zone is generated, accompanied by the moving of powders within and outside of the powder bed. A dome-shape metal structure is formed in the end without noticeable structural defects. In Fig. 2b and Supplementary Video 2, the laser power was increased to 520 W and also exposed for 1 ms. As revealed by the images, the laser-metal interaction is much more violent than the previous case. The Ti-6Al-4V powders and base are quickly melted locally. Then the continuous laser heating causes large cavity depth and strong oscillation behavior, and the molten metal spreads outwards from the surface with a portion getting ejected away. The end structure on the surface is also in a dome shape, but a ~150 µm pore is formed at the bottom of the melt pool deep inside the base.

The competition between the Marangoni convection and the recoil pressure primarily determines the extent of the mass and the heat transfers, as well as the dynamics of the melting process^11, 14, 21, 26^. The extremely high and non-uniform thermal distribution in the melt pool leads to a large surface tension gradient, creating the conditions for Marangoni convection^27, 28^. The surface liquid flows from the center of the laser-heated zone (lower surface tension) to the surrounding regions (higher surface tension), as does the heat. Meanwhile, the absorption of the laser energy results in strong metal evaporation around the laser beam, and the rapid moving of the metal vapor generates recoil pressure gradients and consequent melt ejections^15, 29^. The balance of surface tension and recoil pressure also governs the melting process shown in Fig. 2a, but their collective impact on the melt pool dynamics and powder motion is much higher in the case shown in Fig. 2b.

The pore formation at the bottom of the melt pool in Fig. 2b should be attributed to the relatively large cavity depth^13, 13, 16, 30^. After the laser is turned off, the sudden absence of the laser beam creates a local negative pressure environment, which brings about the liquid metal flowing towards the center of the melt pool^13, 27^. Owing to the large cavity depth, most of the liquid metal that was spread out by the high recoil pressure is maintained at the top level and has a relatively high movability, while that at the bottom level moves too slowly to fill the cavity, which results in the formation of a pore^13^. For the first time ever, the time taken for the closure of the keyhole was measured experimentally, which is about 50 µs in the case shown here. After formation, the keyhole pore tends to float up because of the buoyancy effect. Figure 2b shows that the pore is retained deep inside the base because it fails to reach the top surface of the liquid before being pinned by the solidification front.

In brief, two typical melting modes are revealed here: conduction mode and keyhole mode^13, 21^. The keyhole mode melting occurs when excessive thermal energy density impinges on the sample, such as the case shown in the sequence in Fig. 2b.

#### Melt pool profile

Based on theories and empirical knowledge, many models have been developed for predicting melt pool dimensions^31, 32^, yet the direct observation of the dynamic evolution of melt pool is lacking, particularly its structure underneath the surface^18, 21^. Here, we demonstrate that our high-speed hard-X-ray imaging technique is capable of probing the melt pool development in the LPBF process with sufficiently high spatial and temporal resolutions.

**Figure 3:**
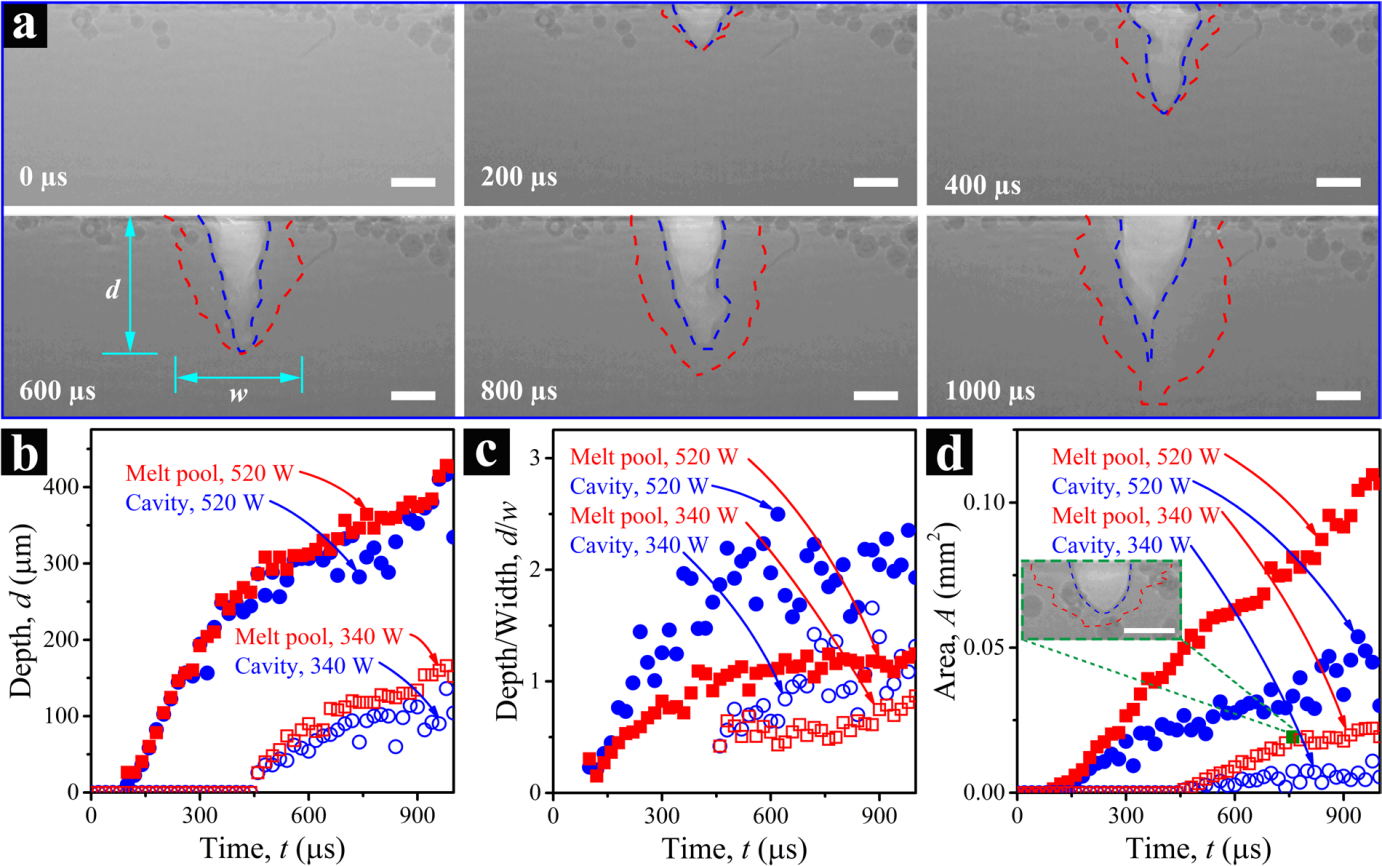
Dynamic evolution of the melt pool in laser powder bed fusion processes of Ti-6Al-4V. (**a**) X-ray images of melt pools in the metal base at *t* = 0, 200, 400, 600, 800, and 1000 µs, respectively. The blue dashed line marks the boundary of the cavity or depression zone, and the red dashed line indicates the liquid-solid interface. The power and size of the laser beam are 520 W and ~220 µm (1/e^2^), respectively. The laser is turned ON at *t* = 0, and continues to heat the sample till *t* = 1000 µs. The powder particle size is in the range of 5–45 µm, and the powder layer thickness is ~100 µm. (**b**–**d**) Development of the melt pool size and geometry as a function of laser heating time under two different laser power conditions: (**b**) depth, (**c**) aspect ratio (depth/width), (**d**) nominal area. The inset to (**d**) shows a steady-state melt pool configuration at the time indicated by the solid green square. All the scale bars in (**a**) and in the inset to (**d**) are 100 µm.

Figure 3a and Supplementary Video 4 show a series of X-ray images of the melt pool during the same LPBF process shown in Fig. 2b. The cavity and the melt pool are outlined using blue and red dashed lines, respectively. Here, only the melt pool structure in the base metal is delineated because the liquid metal in the powder bed can be observed directly without the aid of any image processing. In these images, the cavity or depression zone induced by the recoil pressure appears much lighter in contrast and can be easily recognized. Identification of the outer boundary of the melt pool is more challenging because of the small density difference between liquid and solid Ti-6Al-4V, but this can be accomplished by using a simple image analysis approach described in the Supplementary Information and Supplementary Fig. 3.

Figure 3b–d summarize the dimension change of the melt pool as a function of laser heating time. The depths of the cavity and melt pool for two laser power conditions are shown in Fig. 3b. These show that the higher-power (520 W) laser beam starts to generate the melt pool in the metal base at a much earlier heating stage, and also “drills” much deeper, compared with the lower-power beam (340 W). Under both power conditions, the depth of the cavity is similar to the depth of the melt pool. This is because the bottom of the melt pool has the highest temperature in the sample, which results in the largest recoil pressure, driving liquid metal away from the bottom of the cavity^15, 29^. Figure 3c shows the changes in aspect ratios (i.e. depth/width) of the cavity and melt pool as functions of heating time. The aspect ratio of a melt pool is a parameter commonly used for distinguishing the melting mode^33, 34^. In the case of 340 W laser power (open red square), the depth/width ratio is close to 0.5 at the early stage of heating, meaning that the melt pool is about semicircular in shape (inset to Fig. 3d), which is often regarded as an evidence of conduction mode melting^33^. Beyond *t* = ~750 µs, the aspect ratio starts to increase, indicating a tendency towards the keyhole mode of melting as the thermal energy input continues to increase. When using a higher laser power (solid red square), the depth/width ratio quickly reaches ~1.3 and remains almost unchanged through the laser heating process, indicating stable keyhole mode melting^33^. The aspect ratios of the cavities are higher than the corresponding melt pools in both cases. The width of the cavity is close to the laser beam size through the entire heating process, while the depth continues to increase. Figure 3d plots the areas of the cavities and melt pools as functions of laser heating time. Interestingly, all plots in (b–d) show a clear transition from higher ramp rates to slightly lower rates. Such a transition occurs at *t* = ~450 µs in case of the higher laser power and *t* = ~750 µs for the lower power. We speculate that the increased recoil pressure and Marangoni effect at the later stage of laser heating lead to a strong and complex fluid flow, which helps regulate the sample temperature and maintain a relatively stable melt pool profile.

#### Powder motion

In LPBF processes, powder spatter ejection is commonly observed, and too large a fraction of ejected particles can negatively impact the building process^14, 35, 36^. The ejected powders and molten droplets will fall back down to the powder bed unless removed by, e.g., a gas stream. If these particles fall within the part being built, they will likely contribute to the structural defects in the end product^35^; whereas if they fly far enough from the build area, they may still become an issue for powder recycling by generating agglomerates^35, 36^. Moreover, the formation of a denudation zone near the laser scan path often accompanies powder ejection, which leads to poor build precision and quality^14^. In this contribution, we demonstrate the unique capability of high-speed X-ray imaging technique in studying the powder ejection phenomenon in the LPBF process.

As previously shown in Supplementary Video 2, when a high-power laser impinges on the powder bed, the particles in the laser spot quickly melt and partially evaporate. The metal vapor then “splashes” the nearby particles out of the powder bed. As the melt pool develops, the Marangoni flow and recoil pressure drive the liquid metal flow outwards from the laser beam, wetting and melting particles in the close vicinity upon contact. The circulating liquid flow driven by the surface tension acts to “pull” particles into the melt pool^27^. After a small particle-depleted zone is formed near the laser beam, the particles further away from the melt pool move towards the laser beam, entrained by the Ar gas flow, as the quick expansion of the metal vapor causes the pressure drop around the laser beam due to the Bernoulli effect^15^. The phenomenon of powder flow and ejection were well explained by Matthews and other groups^14, 15^.

In this investigation, based on the fast compressive tracking technique proposed by the Yang group^37, 38^, we developed a set of codes for tracking the motions of particles. Since only 2D images were collected in our experiments, the measured velocity of each individual particle is the projection of true velocity on the 2D plane perpendicular to the incident X-ray direction. Particle motion tracking for the cases of two laser powers are shown in Supplementary Videos 5 and 6. The effectiveness of the tracking algorithm is shown in Supplementary Video 7.

**Figure 4:**
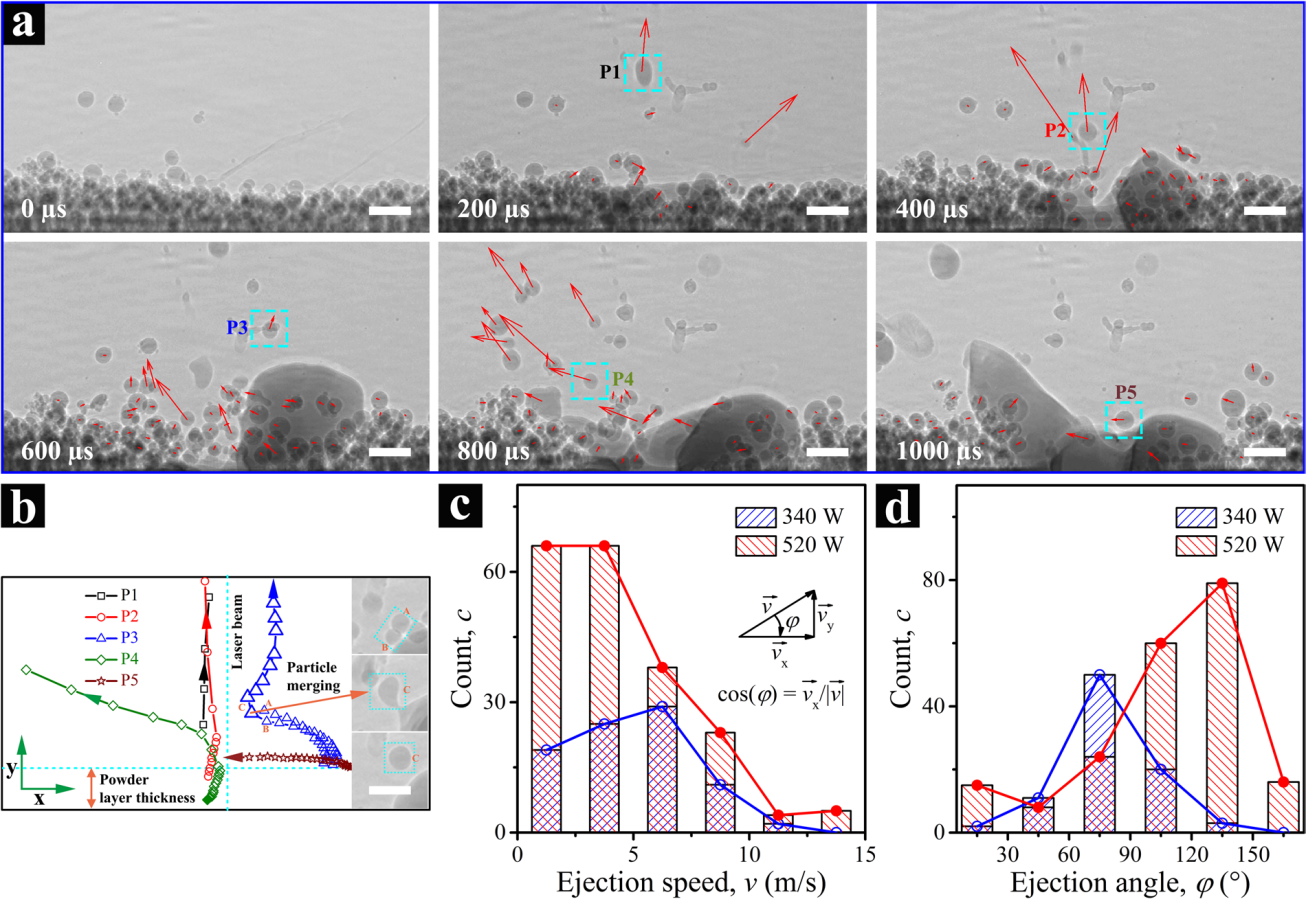
Tracking of powder motion during the laser powder bed fusion process of Ti-6Al-4V. (**a**) Dynamic X-ray images collected at different times, with red arrows indicating the velocity of each individual powder particle. In this case, the laser power and beam size are 520 W and ~220 µm (1/e^2^), respectively. The particle size is in the range of 5–45 µm, and the powder layer thickness is ~100 µm. The full tracking procedure for two laser power conditions are shown in Supplementary Videos 5 and 6. (**b**) Moving trajectories of five different particles P1–P5, which are marked out using cyan dashed squares in (**a**). The dimensions of (**b**) and each image in (**a**) are identical. The vertical and horizontal cyan dashed lines specify the location of the laser beam and the top surface of the powder bed, respectively. The insets of (**b**) show a particle merging process. All the scale bars in (**a**) and (**b**) are 100 µm. (**c**,**d**) Statistics of the powder moving velocity. Distributions of (**c**) ejection speed and (**d**) ejection angle of particles outside the powder bed under the laser power of 340 W and 520 W are shown in blue and red colors, respectively.

Figure 4a shows six images of the case with 520 W laser power. The arrow on each powder particle indicates the direction of motion and the length of the arrow corresponds to the speed. Figure 4b shows the trajectories of five representative particles (marked in Fig. 4a using cyan dashed squares), in which the open symbols mark their locations in consecutive X-ray images, and the arrows point along their directions of motion. P1 is a particle on top of the powder bed, which is ejected immediately by the metal vapor jet at the early stage of the laser heating. P2 could be a particle that is at some distance away from the laser spot along incident X-ray direction. It may have moved towards the laser spot relatively slowly in the beginning but once it reached the laser heating zone, it was ejected out of the powder bed. P3 is the coalescence of two powder particles, as shown in the inset. Two particles (A and B) move towards the laser spot, driven by surface tension and complex metal vapor and gas flow. Once they reach the laser beam, they melt and merge into one particle (C), which is then ejected. P4 is similar to P2, but exhibits a seemingly small ejection angle. P5 is a particle that is entrained into the melt pool at the final stage when the metal vapor flux is much lower, so no ejection occurs. The trajectories of these five particles vividly reveal the complex interplays among the recoil pressure, Marangoni convection, and Ar gas flow.

Figure 4c and 4d show the statistics of the particle ejection speed and the direction under the conditions of two different laser powers, respectively. In both cases, particles outside the powder bed in all X-ray images collected during laser heating (i.e. *t* = 0~1000 µs) are counted. Since the velocity of the same particle may vary with time, we consider the motions in all images to be independent. In Fig. 4c, two major differences can be observed: first, the 340 W laser ejects far fewer particles from the powder bed than the 520 W laser; second, the maximum particle speed in the case of 340 W laser is smaller than that in the higher laser power case (12.3 m/s vs 14.9 m/s). Both results indicate a milder particle motion if the laser power is lower. Figure 4d shows the angular distribution of ejection. The definition of *φ* is shown in Fig. 4c, with *φ* = 90° being the vertical direction, pointing up. In principle, the distribution of angles should be perfectly symmetrical around *φ* = 90° if an adequate number of melting events are counted. However, in a single melting event, the angular distribution of the particle ejection is strongly influenced by the dynamic flow of the melt pool. The complex recoil pressure and surface tension distributions can result in an asymmetric distribution of particle ejection angles, as shown here. A subtle, yet distinguishable, difference between the two laser power cases is that the angular distribution in case of 340 W laser appears more symmetrical around the laser beam direction than the case of 520 W laser, which again is the result of a less turbulent melt pool flow.

Here, we only present a very simple way to gather statistics. Given the high spatial-temporal resolution of the X-ray images and the well-developed feature-tracking algorithms, we believe many other analyses can be done. However, it should be noted that the results from these 2D images work most effectively for validating theoretical models. The missing information along the incident X-ray beam direction means that it is essential to have models to interpret the results.

#### Solidification rate

The solidification in LPBF is a highly localized and extremely fast process owing to the high cooling rate and large thermal gradient^18, 39, 40^. The grain morphology and texture of the end product are strongly affected by the solidification rate, but its quantification has been challenging. Here, we demonstrate the feasibility of measuring the solidification rate in the LPBF process using high-speed hard-X-ray imaging technique.

**Figure 5:**
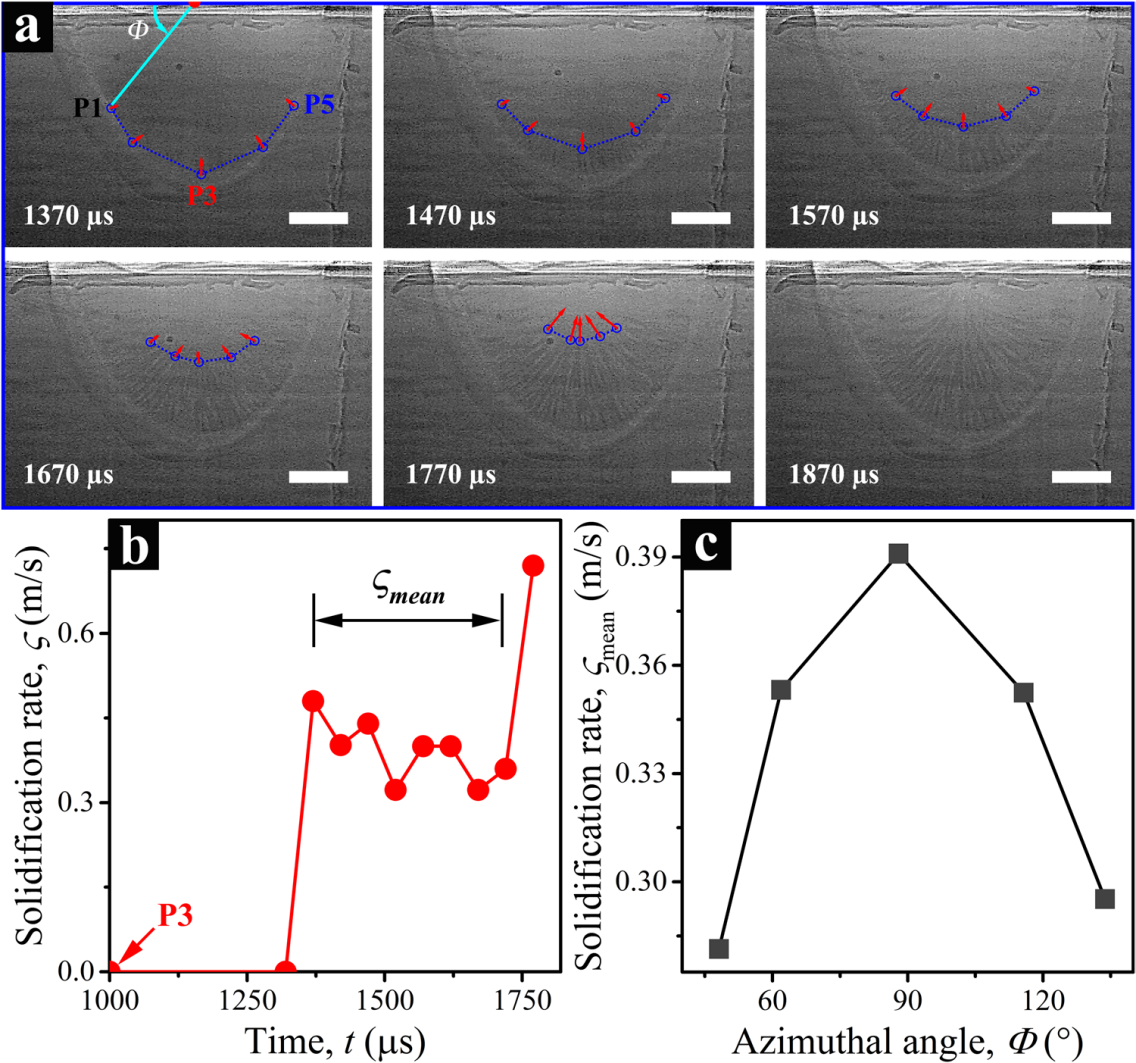
Measurement of the solidification rate of a Ti-6Al-4V plate sample in laser melting process. (**a**) Dynamic X-ray images of the solidification process collected at *t* = 1370, 1470, 1570, 1670, 1770, and 1870 µs. Note the laser was turned off at *t* = 1000 µs. The red dot at the top of (**a**) locates the center of the melt pool. The blue circles are the target points on the liquid-solid interface for tracking, and are connected by blue dotted lines to outline the melt pool boundary. The red arrow on each spot indicates the solidification velocity. All the scale bars are 100 µm. (**b**) Solidification rate of target point P3 as a function of time. The solidification rate is calculated over the time unit of 50 µs. (**c**) Mean solidification rate as a function of the azimuthal angle. The mean solidification rate is averaged over the equilibrium growth period shown in (**b**), and the azimuthal angle is defined in (**a**).

Supplementary Video 8 and Fig. 5a show the solidification process of a Ti-6Al-4V plate sample (~450 µm thickness and no powders on the top), as proof of concept. A near semicircular melt pool can be observed, and the solid-liquid interface is readily identified in the raw images. In Fig. 5a, the solidification front is outlined by the blue dotted line. Five locations (P1–P5) distributed around the solidification front are selected for more quantitative analysis. The red arrow at each location points to the instantaneous solidification direction, and its length corresponds to the solidification rate. The radial growth of columnar grains can be clearly observed, with the long axis aligning well with the thermal gradient in the melt pool^40^. The gradual “curving” growth of these grains reflects the continuous optimization of the growth directions along the local maximum thermal gradients and the easy growth directions. Figure 5b plots the solidification rate as a function of time for the spot P3. When the laser is turned off at *t* = 1000 µs, the dynamic fluid flow in the melt pool does not stop immediately and the melt pool temperature is still higher than the solidification temperature. Detectable solidification occurs at about 350 µs later, with the immediate rate of ~0.48 m/s. With the time lapse, the solidification rate attains a relatively steady state for about 400 µs, but continues to drop. A similar trend is found for data from the other four locations. This slight drop could be caused by the so-called recalescence phenomenon^41–43^. The nucleation of solid phase in the solidification front results in an increase in the local liquid temperature. The thermal gradient then decreases, leading to a drop in solidification rate. At *t* = 1750 µs, the solidification rate increases to ~0.75 m/s as the columnar grains approach the center of the melt pool, which suggests that the local maximum thermal gradients at the center of the melt pool coincide well with the easy growth direction, promoting the most efficient grain growth^40^. Similar growth patterns are found in the other four cases (P1, P2, P4, P5). Figure 5c shows the mean solidification rate at five locations at their equilibrium growth stage (marked in Fig. 5b), as a function of their initial azimuthal angles (defined in Fig. 5a). Evidently, the closer to the centerline of the melt pool, the better the local maximum thermal gradient matches with an easy growth direction, and the faster that solidification proceeds.

#### Phase transformation

Even though the phase transformation behavior of Ti-6Al-4V has been extensively studied^44, 45^ and the time-temperature-transformation (TTT) diagrams have been established^46, 47^, *in situ* characterization of its phase transformation in laser AM has yet to be accomplished. Here, we demonstrate that the highly dynamic phase transformation of Ti-6Al-4V in the LPBF process can be probed using diffraction data from the same high-speed X-ray experiment.

**Figure 6:**
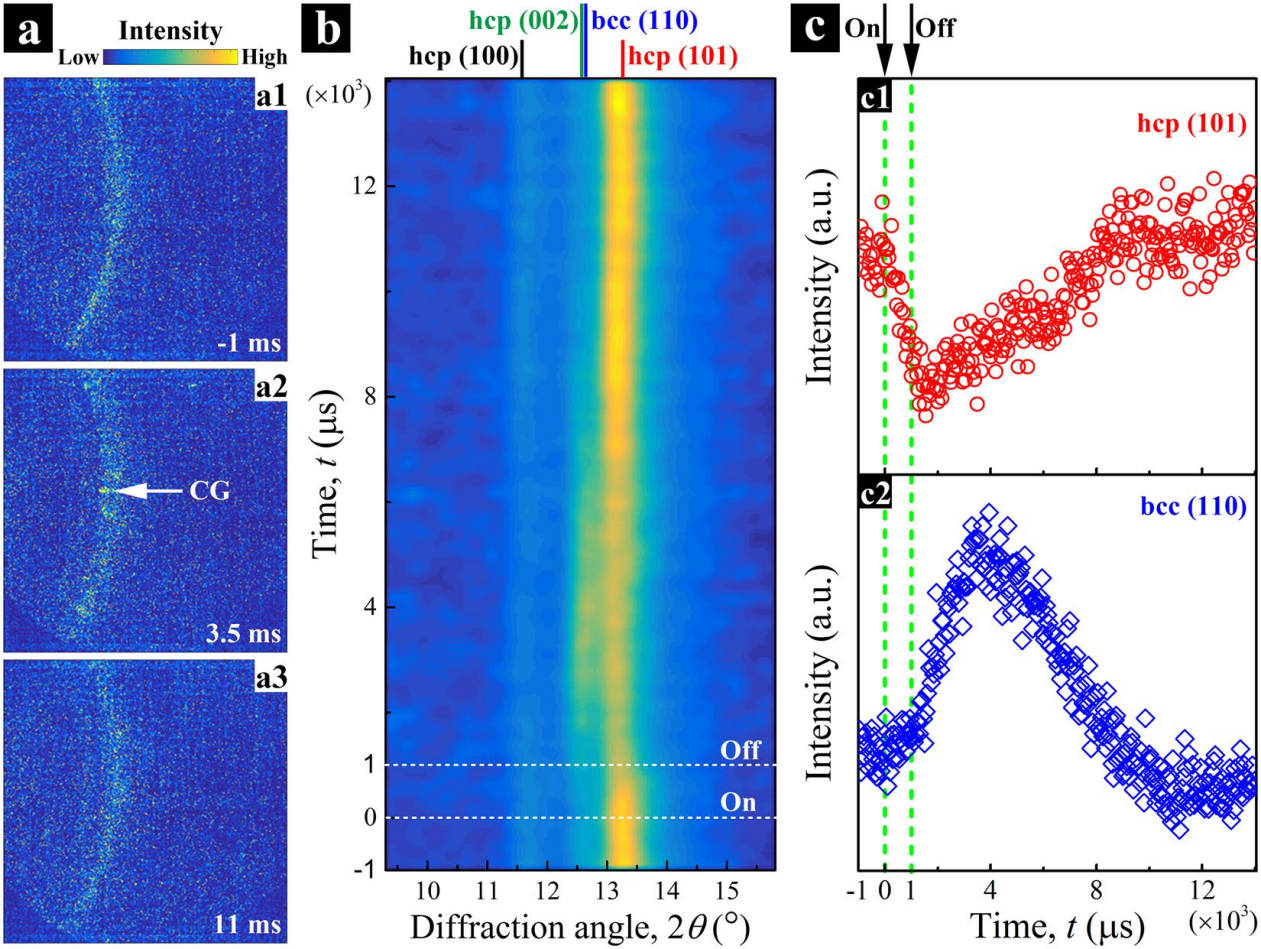
High-speed X-ray diffraction of Ti-6Al-4V in laser melting and solidification process. (**a**) A time sequence of three representative diffraction patterns collected at the time, *t* = −1 ms, 3.5 ms, and 11 ms, respectively. The continuous diffraction rings with uniform intensity distribution in (a1) are a reflection of the starting fine *α* grain structure. The strong diffraction spots that appear in (a2) indicate the formation of coarse *β* grains (CG) in the sample; while the return to continuous rings with uniform intensity distribution in (a3) indicates a transformation back to a finer grain microstructure. (**b**) Time-resolved diffraction intensity map. (**c**) Diffraction intensities of hcp-(101) and bcc-(110) peaks. The dashed lines in (**b**) and (**c**) marked the start and end of laser heating.

Supplementary Video 9 shows a series of time-resolved diffraction patterns collected during the solidification process of a Ti-6Al-4V plate sample. The size of the X-ray beam is slightly larger than the entire melt pool, as for the case shown in Fig. 5. Figure 6a shows a time sequence of three representative diffraction patterns collected at different stages of the melting and solidification process. A noticeable change in the diffraction patterns is the appearance of strong diffraction spots during the initial solidification and their disappearance as the sample continues to cool down. This is consistent with the established phase transformation sequence for Ti-6Al-4V, such that coarse grains (CG) of the high-temperature *β* phase (bcc lattice) grow out of the melt pool first and then transform into a finer grain microstructure consisting of a two-phase *α*/*β* or martensitic *α* ^*’*^ in the end^48, 49^. Figure 6b summarizes the diffraction results into a time-resolved 2D intensity map, which was constructed by accumulating all the radially integrated 1D intensity profiles. Intensity calibration was applied to the raw data by considering the decrease in the efficiency of the diffraction detector with increasing frame number. The corresponding atomic planes of the *α* (or *α* ^*’*^) phase and *β* phase are indexed at the top of the figure. The dashed lines labeled with “ON” and “OFF” mark the start and end of the laser heating. The changes in intensity for the different diffraction peaks reflect the melting/solidification and phase transformation behaviors of the sample, while the shifts in peak positions correspond to the expansion and contraction of the crystal lattice during heating and cooling.

The graphs in Fig. 6c show the diffraction intensities of the hcp-(101) and bcc-(110) peaks as functions of time. As the laser heating starts, the hcp-(101) intensity peak starts to drop immediately, while the bcc-(110) peak increases slightly, suggesting that the *α* → *β* phase transformation is a slow process that is incomplete before the solid phase melts. When the laser is turned off, the hcp-(101) intensity peak continues to decrease, implying that some regions of the sample (e.g., the base surfaces adjacent to the outer edges of melt pool) continue to melt due to internal heat transfer. The hcp-(101) peak reaches a minimum value at *t* = ~1500 μs. It then starts to increase with time until *t* = ~9000 μs, and remains almost constant afterwards. On the other hand, the bcc-(110) diffraction intensity shows a peak at *t* = ~4000 μs, indicating the growth of the *β* phase and its transformation to the *α* (or *α* ^*’*^) phase as the temperature drops below the *β*-transus (*β*_tr_, ~994 °C). According to the previous reports^46, 50^, the Martensite start (*M*_s_) temperature is 575 °C, and with a sufficiently high cooling rate (above 410 °C/s), Ti-6Al-4V will experience a fully diffusionless transition from the *β* phase to the *α* ^*’*^ phase. Here, the cooling rate is conservatively estimated as ${v}_{c}=({T}_{m}-{M}_{s})/{\rm{\Delta }}{t}_{c}$, where *T*_m_ is the melting point of Ti-6Al-4V (~1650 °C), and Δ*t*_*c*_ is the time needed to complete the transformations from the liquid metal to the hcp phase. From Fig. 6c, Δ*t*_*c*_ is estimated to be 10^4^ μs, from the time when the laser is turned off (*t* = 1000 μs) to the full transition from the bcc phase to the hcp phase (*t* = ~11000 μs). Hence, the lower bound of the cooling rate is about 10^5^ °C/s, much higher than the required 410 °C/s, suggesting that the transformation product is *α* ^*’*^ martensite. Another interesting observation from Fig. 6c is that the minimum diffraction intensity of hcp-(101) and the maximum intensity of bcc-(110) do not present at the same time. The early onset of an increase in the hcp-(101) intensity indicates an extremely fast phase transformation process. The transformation rate is conservatively estimated as ${v}_{g}={d}_{m}/{\rm{\Delta }}{t}_{g}$, where *d*_*m*_ is the depth of the melt pool and Δ*t*_*g*_ is the time needed to complete the solid phase transformation. Obviously, Δ*t*_*g*_ < Δ*t*_*c*_ < 10^4^ μs. Here, the depth of the melt pool is on the order of 10^2^ µm. Thus, the phase transformation rate is on the order of 10^4^ µm/s, about two orders higher than the reported values for the *β* → *α* ^*’*^ massive (diffusionless) transformation^51, 52^. This substantial increase is consistent with the high cooling rates obtained with laser melting and sub-millimeter melt pools^40, 46, 50^.

## Conclusion

In summary, we present here the application of high-speed X-ray imaging and diffraction techniques for *in situ* characterization of the metal LPBF process. As demonstrated above, many important physical processes involved in LPBF of Ti-6Al-4V, including melt pool dynamics, powder ejection, rapid solidification, and phase transformation, can be studied quantitatively with unprecedented spatial and temporal resolutions. In particular, a few critical phenomena are experimentally observed and quantitatively measured. First, the entire formation process of the keyhole pore deep inside the base metal is revealed in considerable detail using high-speed X-ray imaging. It is found that the closure of the keyhole takes less than 50 µs, and the voids generated deep inside the material are pinned by the advancing solidification front. Secondly, the dimensions of the melt pool as a function of the laser heating time are carefully measured. During the growth of the melt pool, a transition from a higher growth rate to a lower rate can be observed, indicating a strong and complex flow of the molten metal at the later stage of laser heating, which tends to moderate the sample temperature and maintain a relatively stable melt pool profile. Thirdly, a suite of codes are developed to track the motions of Ti-6Al-4V powder particles. The maximum ejection speed, in the case of high thermal energy input, could be as high as 15 m/s. Fourthly, under the conditions in the present work, the spatially and temporally averaged solidification rate of Ti-6Al-4V is about 0.4 ~ 0.5 m/s. A slight decrease in the solidification rate during the steady growth stage is observed, which likely arises from the recalescence behavior of solidifying material. Finally, the phase transformation of Ti-6Al-4V is probed by the high-speed X-ray diffraction technique. By examining the evolution of the intensities of the hcp-(101) and bcc-(110) diffraction peaks with cooling time, a transformation rate above 10^4^ µm/s can be estimated, suggesting that a diffusionless *β* → *α* ^*’*^ phase transformation occurs.

We believe that the experimental and data analysis approaches presented here open a new chapter for the research and development of AM, as well as other laser processing and manufacturing, such as welding. The results from the high-speed X-ray experiments will help us not only understand the physics underpinning the formation of different defects, but also build high-fidelity models to guide the process optimization for manufacturing parts with different geometries and dimensions. Looking back, metal AM has experienced a rapid development in recent years, thanks to the substantial investment in the technology from both public and private sectors worldwide. However, a precise control of microstructures and properties of additively manufactured products based on a fundamental understanding remains challenging. Also, development of new materials suitable for additive manufacturing and new techniques for fabricating multi-material products has only just begun. Undoubtedly, *in situ* structure characterization techniques, such as those we described here, will become a critical element in the field of AM, and help scientists and engineers solve long-standing problems and exploit new opportunities.

## Methods

### Materials

Ti-6Al-4V powders (EOS *Titanium Ti64*, EOS GmbH, Germany) and plates (Grade 5, McMaster-Carr, USA) are studied. The powders, designed for the commercially available EOS LPBF machines, are of spherical shape, and the particle size is in the range of 5–45 µm. The thin plates, acting as the base for the powders, have an average thickness of 450 µm, and a height of 3.0 mm. For the Ti-6Al-4V plates, the density is 4.43 g/cm^3^, the thermal conductivity is 6.9–7.3 W/(m∙K) at 294 K, and the melting point is ~1650 °C.

### Vacuum chamber and powder-bed system

The custom-built vacuum chamber is made of stainless steel. On the top, there is a laser entrance window, made of fused silica. The laser beam transmits through this window, enters into the chamber and interacts with the miniature powder bed sample. The X-ray beam enters and exits the chamber via two Kapton windows. There are a few ports on the chamber for observation, pumping, infilling gas, and mounting feedthroughs for electronic control and feedback. Ar gas is filled into the chamber to prevent the potential oxidation of the metals. The miniature powder bed sample consists of two identical pieces of glassy carbon (vitreous) plates (Grade 22, Structure Probe Inc., USA) with the thickness of 1.0 mm and the height of 6.0 mm. The gap width between the two plates is determined by the thickness of the Ti-6Al-4V base. On top of the metal base, one layer of Ti-6Al-4V powders with the layer thickness of ~100 µm is spread out evenly. The sample, laser beam and X-ray beam are aligned using a set of step motors.

### Laser system

The laser system is equipped with an ytterbium fiber laser (IPG YLR-500-AC, USA) and a laser head (IPG FLC 30, USA). The fiber laser is in single-mode, providing pure Gaussian beam profiles. The wavelength is 1070 nm and the maximum power is 520 W. The fiber laser can be operated in both CW mode and modulation mode (rate up to 50 kHz). In this study, the laser emission is controlled by a “gating” signal sent by a delay generator (SRS D535, Stanford Research Systems, USA), and the duration is 1000 µs. At the focal spot, the laser beam size is 50 µm. In the experiments, the sample is positioned 7 mm away from the focal point to achieve a ~220 µm spot size (1/e^2^, Gaussian beam).

### High-speed synchrotron X-ray imaging and diffraction

As illustrated in Fig. 1, a short-period (1.8 cm) undulator with the gap set to 12 mm generates polychromatic X-rays with the first harmonic energy at 24.4 keV (*λ* = 0.508 Å). A pair of slits were used to define the size of the X-ray beam. The imaging detection system is composed of a LuAG:Ce scintillator (100 µm thickness), 45° reflection mirror, a relay lens, an objective lens (Edmund Optics Inc., Barrington, NJ), and a high-speed camera (Photron FastCam SA-Z, USA). The diffraction detection system integrates a LYSO scintillator, a Quantum Leap image intensifier (Stanford Computer Optics Inc., Berkeley, CA) and a high-speed camera (Photron FastCam SA-Z, USA). The diffraction data is analyzed using a home-developed software: *HiSPoD* ^24^.

## Electronic supplementary material

Video S1

Video S2

Video S3

Video S4

Video S5

Video S6

Video S7

Video S8

Video S9

Supplementary Materials
