# Supplementary material for: Real-time monitoring of laser powder bed fusion process using high-speed X-ray imaging and diffraction
Source: Sci Rep. 2017 Jun 15;7:3602. doi: 10.1038/s41598-017-03761-2 (PMC5472560; doi:10.1038/s41598-017-03761-2)
Supplement: Supplementary file 10 — Supplementary Materials [file 41598_2017_3761_MOESM10_ESM.pdf]

Supplementary Material for

Real-time monitoring of laser powder bed fusion process using high-speed X-ray imaging and diffraction

Cang Zhao[1], Kamel Fezzaa[1], Ross W. Cummingham[2], Haidan Wen[1], Francesco De Carlo[1], Lianyi Chen[3], Anthony D. Rollett[2], Tao Sun[1,*]

1. X-ray Science Division, Advanced Photon Source, Argonne National Laboratory, Argonne, IL 60439
2. Department of Materials Science and Engineering, Carnegie Mellon University, Pittsburgh, PA 15213
3. Department of Mechanical and Aerospace Engineering, Missouri University of Science and Technology, Rolla, MO 65409

* Corresponding Author: *taosun@aps.anl.gov*

Table of Contents

Quantifications of laser powder bed fusion process 2

1. Determination of melt pool profiles 2

2. Particle tracking 2

3. Determination of solidification rate 3

Supplementary Figures 4

Supplementary Videos 7

References 8

Quantifications of laser powder bed fusion process

1. Determination of melt pool profiles

For the melt pool, as presented in Fig. 2, it can be recognized easily above the metal base; however, below the base surface, it becomes fairly difficult to detect directly through human eyes. In order to show the melt pool dynamics clearly, particularly in the region of interest below the base surface, we developed a set of algorithm codes based on the intensity profile of each horizontal line on the X-ray images. First, as illustrated in Supplementary Fig. 3b, the X-ray image at the time of t was divided by the background image at the time of zero to enhance the features of interest. Second, in Supplementary Figs. 3c and 3d, around the laser beam, the local peak locations of the second derivation of the intensity profile on a horizontal line (Supplementary Fig. 3b) were marked out by blue dashed lines and blue empty circles to show the boundaries of the cavity (C) and the liquid phase (L). Third, the locations of the intensity profile, where the intensity values equal the background intensity (red dashed horizontal line in Supplementary Fig. 3c2), were labeled by red dashed lines and red solid circles to indicate the boundaries of the liquid phase and the solid phase (S). Eventually, as shown in Supplementary Fig. 3d, a blue dashed line was used to connect the blue empty circles on all the horizontal lines and were smoothened to show the C-L interface; a red dashed line was employed to connect the red solid circles on all the horizontal lines and were also smoothened to show the L-S. Based on the C-L and the L-S interfaces, the nominal areas (A) of the cavity and the liquid phase at the frame time of t are estimated as

$$A = \sum_{i=1}^{n} D_i \cdot \Delta h \qquad (1)$$

where D_i is the length between the two intersection points of the C-L or the L-S interface over the ith horizontal line of an X-ray image, Δh is the height interval between the neighboring horizontal lines, and n is corresponding to the maximum penetration depth (Fig. 3b, $d = n \cdot \Delta h$). The calculation results of nominal areas are summarized in Fig. 3d. In addition, the maximum melt pool widths (w) were measured, and the ratios of d/w are shown in Fig. 3c, as a function of the frame time, t.

2. Particle tracking

The fast compressive tracking technique proposed by the Yang group provides an efficient solution for tracking a moving object in real-time [1]. In this study, we modified and improved the technique to accommodate our applications of tracking the particle motions accurately through a set of X-ray images. First, from the original images (e.g. Fig. 4a), the locations of the targeted particles (e.g. P1-P5) were marked out by the Matlab built-in function of imfindcircles and were confirmed by human eyes. Second, the X-ray images were enhanced through the Local Equalization function built in the Image-Pro Plus software (Media Cybernetics Manufacturing, Warrendale, PA) to distinguish the targeted particles from their backgrounds [2]; the enhanced local backgrounds also provided better references for detecting the targeted particles. Third, the algorithm codes were run to track the trajectory of each targeted particle (e.g. P1-P5 in Fig. 4b), and the results were confirmed by human eyes. As an example, the particle tracking process was illustrated in Supplementary Video 7. Among all the particles outside the powder bed, the ejection speeds and angles were statistically analyzed, and the results are shown in Figs. 4c and 4d, respectively.

3. Determination of solidification rate

After the laser fusion process, the melt pool started to cool down, and columnar grains grew along the radial direction. In Fig. 5a and Supplementary Video 8, we chose five points of interest (P1-P5) on the L-S interface, and tracked their motions. The solidification rate, ς, is estimated as

$$\varsigma = \frac{\Delta l}{\Delta t} \tag{2}$$

where Δt is the time unit (50 μs), and Δl is the corresponding moving distance of the targeted point on the L-S interface. Fig. 5b shows typical history of the solidification rate of a targeted point (e.g. P3). Over the time spanning the plateau in Fig. 5b, the solidification rate was averaged, and the results are shown in Fig. 5c.

Supplementary Figures

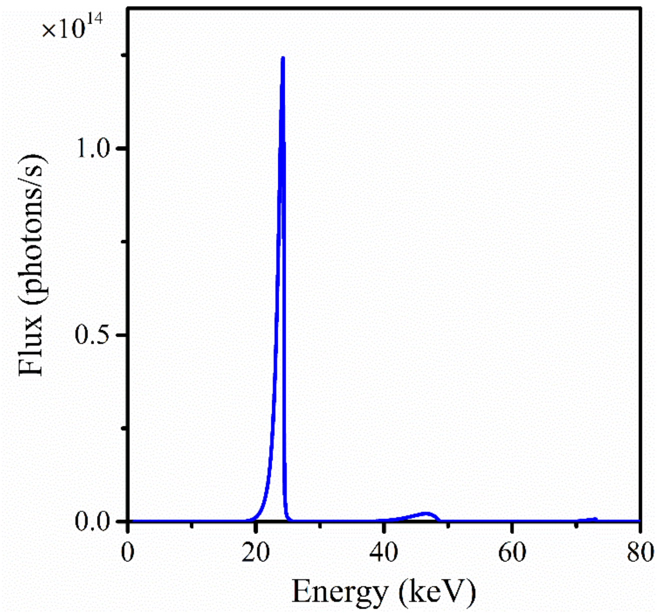

Figure S1. Energy spectrum of X-rays generated by the undulator with a period of 1.8 cm at the 32-ID beamline of the Advanced Photon Source. The gap of the undulator is set to 12 mm. The first harmonic energy is 24.4 keV.

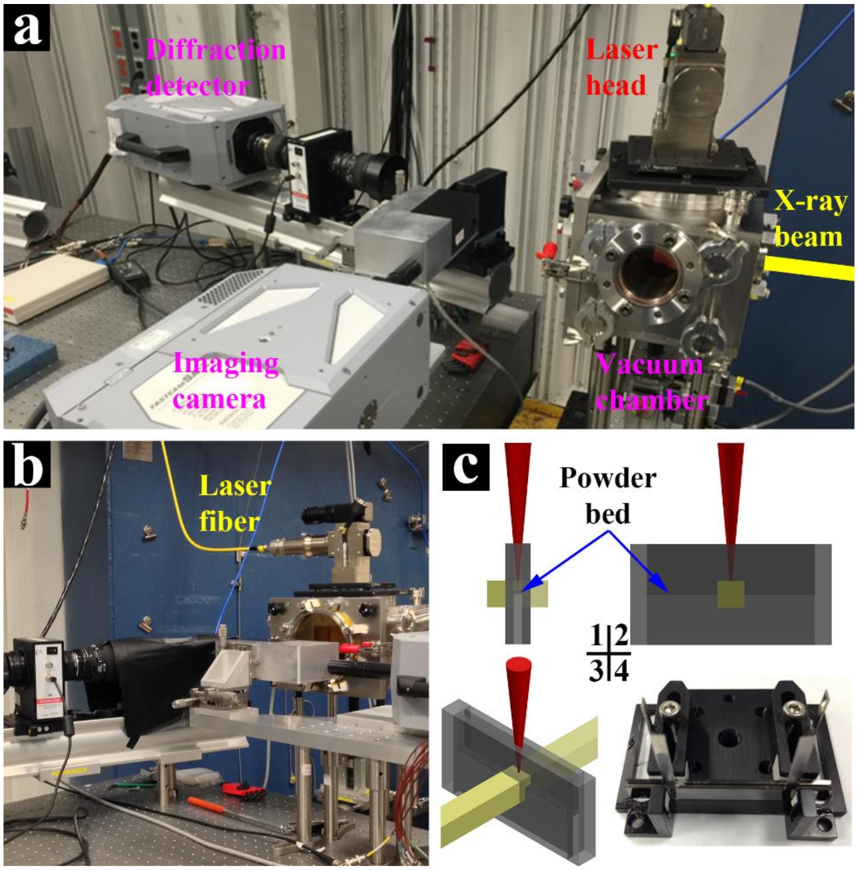

Figure S2. Experimental setup for high-speed synchrotron X-ray experiments on laser powder bed fusion process. (a-b) Photos of the setup from different angles. (c) The powder bed sample, with (c1-c3) showing different views in sketch drawing (X-ray beam is depicted in yellow and laser beam in red) and (c4) showing the photo.

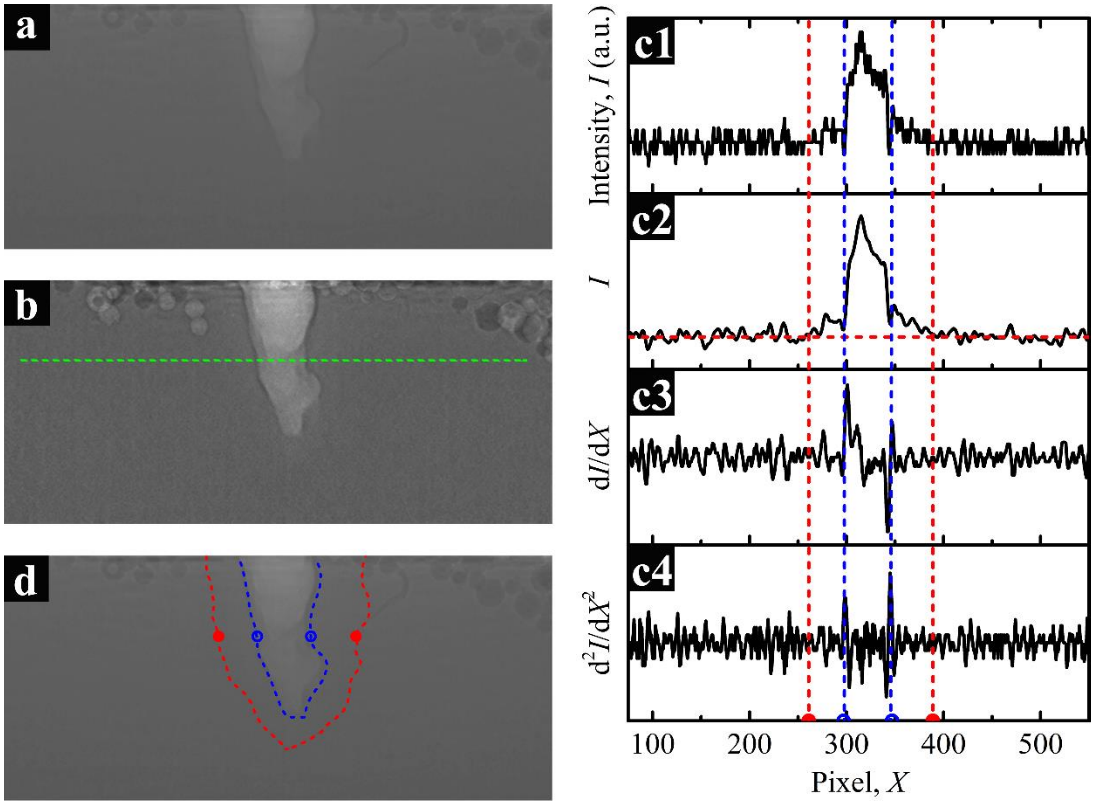

Figure S3. Image analysis algorithm for identifying the cavity and melt pool. (a) Original X-ray image showing the melt pool configuration in the Ti-6Al-4V base. (b) Treated X-ray image, which is divided by the image collected at t = 0 (no melting occurs). (c) 1D image intensity profile (linecut shown in (b) in green dashed line) and its operations: (c1) original intensity profile; (c2) smoothened intensity profile, with the red dashed horizontal line marking the background intensity; (c3) first derivation of the smoothened intensity profile; (c4) second derivation of the smoothened intensity profile. The vertical blue and red dashed lines indicate the boundaries of the cavity and the melt pool, respectively. (d) X-ray image with the boundaries of the cavity (blue) and the melt pool (red) overlaid.

Supplementary Videos

Video S1. High-speed X-ray imaging of laser powder bed fusion process of Ti-6Al-4V with 340 W laser power.

Video S2. High-speed X-ray imaging of laser powder bed fusion process of Ti-6Al-4V with 520 W laser power.

Video S3. Melt pool dynamics in the Ti-6Al-4V metal base with 340 W laser power.

Video S4. Melt pool dynamics in the Ti-6Al-4V metal base with 520 W laser power.

Video S5. Powder motion tracking with 340 W laser power.

Video S6. Powder motion tracking with 520 W laser power.

Video S7. Particle tracking using modified fast compressive technique.

Video S8. High-speed X-ray imaging of the rapid solidification process of Ti-6Al-4V.

Video S9. High-speed X-ray diffraction of laser powder bed fusion process of Ti-6Al-4V.

References

1. Zhang, K., Zhang, L. & Yang, M.-H. Fast compressive tracking. *IEEE Transactions on Pattern Analysis and Machine Intelligence* **36**, 2002-2015 (2014).

2. Zhao C, Qiao Y. Characterization of nanoporous structures: from three dimensions to two dimensions. *Nanoscale* 2016, **8**(40)**:** 17658-17664.
